# Emerging Digital Practices Supporting Student-Centered Learning Environments in Higher Education: A Review of Literature and Lessons Learned from the Covid-19 Pandemic

**DOI:** 10.1007/s10639-023-11789-3

**Published:** 2023-05-20

**Authors:** Sofie Otto, Lykke Brogaard Bertel, Niels Erik Ruan Lyngdorf, Anna Overgaard Markman, Thomas Andersen, Thomas Ryberg

**Affiliations:** Aalborg University, Aalborg, Denmark

**Keywords:** Digital practices, Covid-19/post-pandemic, Student-centered learning, Systematic literature review, Case study

## Abstract

The aim of this paper is two-fold: firstly, to provide an overview of emerging digital practices that support collaborative learning, competency development, and digital literacy for student-centered learning environments in higher education during the rapid digital transition caused by pandemic-related lockdowns across the world, and secondly, to analyze and discuss how systematic reviews of generalized themes and trends can be combined with contextualized experiences and the lessons learned from the Covid-19 crisis to inform the digital transformation of higher education, with a particular focus on bridging the gap between campus-based teaching and online learning and on the identification of the digital competencies that teachers and students must acquire during the continuing shift into a ‘new normal’ for post-pandemic educational practices. This study was motivated by questions and findings emerging from an early reactive case study conducted by three of this paper’s co-authors (Lyngdorf et al., 2021a). By reviewing the full texts of 18 articles, this study provides a systematic literature review which maps the general landscape of the online, hybrid, and blended digital practices applied in existing student-centered learning environments in higher education since the onset of the pandemic. Furthermore, this mapping is used to revisit data and findings from the earlier reactive study of emerging digital practices in a specific problem- and project-based learning (PBL) environment. This study’s findings highlight critical factors and barriers related to emerging practices which support students’ interactions with teachers, content, and each other, as well as the emerging competencies that these practices will require. The paper concludes with a discussion of the main findings and their implications for further research and practice.

## Introduction

The COVID-19 pandemic substantially disrupted established campus-based teaching and learning practices in higher education around the world. Almost overnight, institutions were forced to rapidly adapt to new modes of delivering online and hybrid teaching in order to ensure educational consistency while complying with governmental restrictions. In the past few years, educational systems have undergone various different iterations of both full and partial lockdowns and re-openings, and thus different variations of online, hybrid, and blended modes of teaching. Although the concept of distance learning (DL) is not new (Valentin, 2002; Bayne et al., 2014), the pace and urgency of the transition from face-to-face learning to exclusively online environments has been unprecedented. As such, the phenomena of emergency remote teaching (ERT) (Hodges et al., 2020) has emerged in preliminary reports and research covering digital practices during the pandemic to encompass the improvised, often technology-driven, arrangements that were quickly developed to ensure the delivery of teaching. Inevitably, the crisis has also afforded new digital practices and necessary pedagogical innovations, reigniting an ongoing conversation about quality in education and the need to rethink higher education and teaching in order to integrate tools and methods that foster more active, flexible, and meaningful learning (Rapanta et al., 2021).

To ensure the documentation of contextualized knowledge and experiences from the pervasive digital transformation, we, along with many others, conducted a localized empirical study in the early stages of the first lockdowns in order to explore experiences of student-centered and competency-focused learning environments transitioning into exclusively online teaching (Lyngdorf et al., 2021a, b). This study thus takes as its point of departure a case study situated in a systemic problem-based learning (PBL) environment with a long tradition of practicing PBL at the curriculum level, rooted in pedagogical principles such as group-based and collaborative learning, exemplarity, and authentic problems as the starting point of the learning process (Kolmos & Graaff, 2003). Key aspects of this model have been found to be particularly useful for supporting the development of students’ competencies to allow them to handle disruptive changes in their learning environments as well as in their daily lives.

Subsequent case studies and literature reviews have discussed the numerous new practices and technologies adopted in early reactive studies (Bond et al., 2021; Khan, 2021). Many of these papers have emphasized the need for attention towards aspects inherent to student-centered learning environments, such as student motivation, interaction, engagement, and self-efficacy (Crawford et al., 2020; Hodges et al., 2020; Aguilera-Hermida, 2020) and teacher readiness (Scherer et al., 2021). However, research has yet to provide a systematic review and mapping of these emerging digital practices in student-centered, collaborative, and competency-focused learning environments.

Thus, the aim of this paper is two-fold. The first aim is to provide an overview of emerging digital practices that support collaborative learning, competency development, and digital literacy in student-centered learning environments in higher education during the rapid digital transition caused by lockdowns across the world. The second is to analyze and discuss systematic reviews of generalized themes and trends that can be combined with contextualized experiences and lessons learned from the crisis to inform the digital transformation of higher education, with a particular focus on bridging the gap between campus-based teaching and online learning and on identifying the digital competencies required by teachers and students during the continuing shift into a ‘new normal’ for post-pandemic educational practices.

In the following section, we will introduce the early reactive case study and discuss its research methods and main findings as the basis for the research questions which guide the systematic literature review and scope of the present study. Section 3 will then outline the methodological approach of the systematic review and elaborate on the connections between the literature review and the revisited case study. Section 4 presents findings from the review and the subsequent revisiting of the case study from the perspective of the themes emerging from these findings. Finally, Sect. 5 discusses the implications of these findings as well as the potential and challenges of conducting research in a field undergoing accelerating transformation. Section 6 concludes this paper and provides suggestions for future work.

## Background: conducting exploratory research in a time of crisis

At the onset of the pandemic and during the subsequent lockdowns, many educational researchers across the globe began to conduct ongoing research gathering knowledge of and experiences with ERT in different educational contexts, including K12 and higher education, to continuously inform and develop both research and practice (Jandríc et al., 2020, 2021, 2022; Graham 2022; Georgsen, 2021). One such study was an extensive qualitative case study on digital practices and ERT in a specific student-centered learning environment with problem- and project-based learning as the curriculum model at its core across all faculties and departments. In this particular context, students generally spend half their time each semester (15 out of 30 ECTS) in semester-long group-based project work, and each group (usually consisting of 4–7 students) is assigned a supervisor. Thus, most teachers spend a significant amount of their teaching time supervising students. The case study sought to explore experiences and practices related to these new online and hybrid modes of teaching and learning in an otherwise mostly campus-based, student-centered learning environment. It is important to note that restrictions varied throughout the Covid-19 pandemic: the nation-wide lockdown in the spring of 2020 was significantly eased in the summer of 2020, and students were allowed to be physically present on campus in their group rooms and workspaces as well as in larger clusters without social distancing during lectures for most of the fall of 2020. However, in December of 2020 the number of Covid-19 cases increased significantly, and a campus-wide lockdown was once again implemented to prevent the spread of the virus. This meant students and teachers worked from home during the winter and for the entirety of the 2021 spring semester. Thus, students’ experiences of interacting with teachers/supervisors, fellow students, and digital content varied significantly between 2020 and 2022 depending on the specific lockdown rules in place at the time. Naturally, this is reflected in the data collected from August 2020 to November 2020.

### Case study approach and findings

The case study applied an inductive and qualitative approach to data collection in order to ensure the collection of in-depth and nuanced student and teacher perspectives on their experiences with digitally supported PBL during the first lockdown in the spring of 2020, and into the re-opening with restrictions in the fall of 2020. In the study, 22 focus group interviews were conducted with 60 teachers and 35 students (15 interviews with four teachers each, and seven interviews with five students each) representing 16 departments across all faculties. Particular attention was paid to ensuring representation across educational levels, national and international student and staff backgrounds, and different conditions for participation in online learning (e.g., whether or not the student or teacher had children living at home). Each interview lasted one hour and was conducted online (using MS Teams) or in-person when restrictions allowed for this. The interviews were then transcribed and coded systematically and iteratively in NVivo using thematic analysis (Braun & Clarke, 2006) to apply an inductive approach to the patterns and themes emerging from the dataset. Through the initial thematic analysis, 34 codes were identified, revised, and condensed into a final total of 31 codes. These codes were related to themes such as digitally supported teaching and active learning, project collaboration and supervision, as well as organizational matters, such as organizational barriers and management issues, teachers’ workloads and work-life balance, and digital content rights, among other issues.

Findings from the case study showed, that both teachers and students, being accustomed to a learning environment characterized by on-campus, active, problem-based and collaborative learning in a systemic PBL educational model, were largely dissatisfied with the transition to ERT that dominated the first lockdown. Despite this, ERT was still widespread among teachers and was in some cases preferred to more active and demanding learning approaches such as flipped classroom by some students in the fall of 2020 when restrictions were lifted temporarily (Lyngdorf et al., 2021a, b). This was explained by a lack of digital competence among both groups and limited organizational resources and investments to properly develop, facilitate, and support new digital/hybrid and active learning experiences.

However, the study also found that some of the core elements of the PBL university model did prove agile and resistant to many of the challenges presented by the lockdown. Students were able to sustain a viable level of social interaction supporting the general well-being of students as well as socio-cultural learning processes. Furthermore, the student-centered educational culture of PBL meant that students were still highly self-directed and took responsibility for their own learning by identifying and adapting to the new situation supported by their supervisors, who made an extra effort to make themselves available to students as needed. This allowed for highly contextualized and contemporary project work addressing complex and authentic discipline-specific problems imposed by the pandemic. Whereas both students and teachers reported challenges related to communication, conflict management, and the increasing individualization of the otherwise collaborative learning process, the supervisors were generally quite impressed with students’ ability to adapt to this new norm. Students in later semesters seemed particularly resilient and well-equipped to adjust to the problems they were addressing, their data collection methods, and their collaboration practices, while also inventing new ways to support each other.

Whereas the study highlighted the issues, potential, and barriers involved in digitally transforming PBL during a time of crisis and restrictions, the study was naturally highly contextualized and exploratory. Thus, the findings are not necessarily generalizable or comparable across other student-centered learning environments. Consequently, the purpose of this paper is to provide a systematic overview of research on emerging digital practices that support collaborative learning, competency development, and digital literacy in student-centered learning environments in higher education as reported during the rapid digital transition caused by the global lockdowns, and to revisit the case study in light of the themes emerging from the review.

## Methodology

**Fig. 1:**
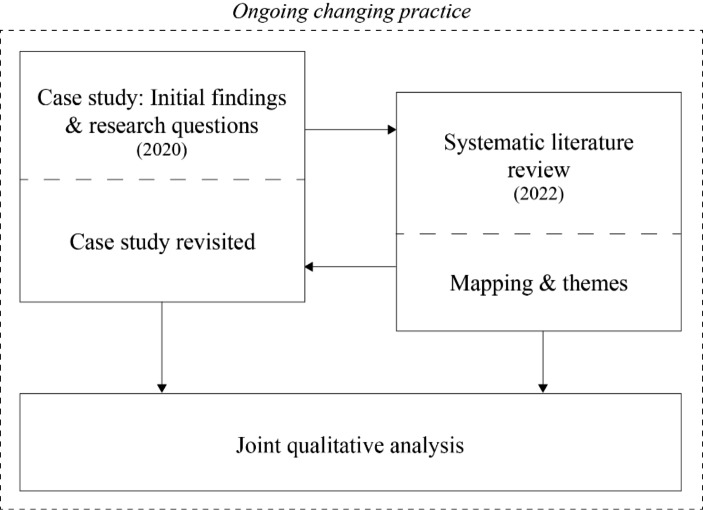
Visualization of methodological approach

This paper builds upon two main methodologies: (1) analysis of the case study presented in Sect. 2; and (2) a systematic literature review guided by the research questions raised on the basis of the case study. Figure 1 visualizes the connections between these two methods. Findings from the systematic literature review and case study will be combined in a joint analysis and discussion, highlighting critical factors and barriers as well as implications for post-pandemic student-centered educational practices in the continuous shift into a ‘new normal.’

### Systematic literature review

A systematic literature review was conducted following the PRISMA 2020 guidelines (Page et al., 2021). A search string was built around three blocks focusing on the following: (1) student-centered learning models and approaches; (2) variations of digital practices and technologies; and (3) literacy and competencies. In each block, we attempted to include as many keywords as possible to cover the relatively broad fields, especially in relation to block 1 and 2, and as a result the search terms shown in Table 1 were included in each block.

**Table 1:** Included search terms in each block

Block	Search terms
1	PBL OR PJBL OR PPL OR “problem based learning” OR “project based learning” OR “problem oriented project learning” OR “inquiry based learning” OR IBL OR “challenge based learning” OR CBL OR “discovery learning” OR “discovery based learning” OR “reflective practice based learning” OR RPL OR “student centered learning” OR “collaborative learning” OR “experiental learning” OR “experiental teaching” OR “cooperative learning” OR “active learning” OR “constructivist teaching” OR “constructivist learning”
2	online OR virtual OR digital OR technolog* OR remote OR distance OR flipped OR ICT OR hybrid OR blended OR “e-learning” OR web OR “learning management system*” OR LMS OR videoconferenc* OR VLE
3	literacy OR competenc* OR “21st century skills” OR “computational thinking”

**Fig. 2:**
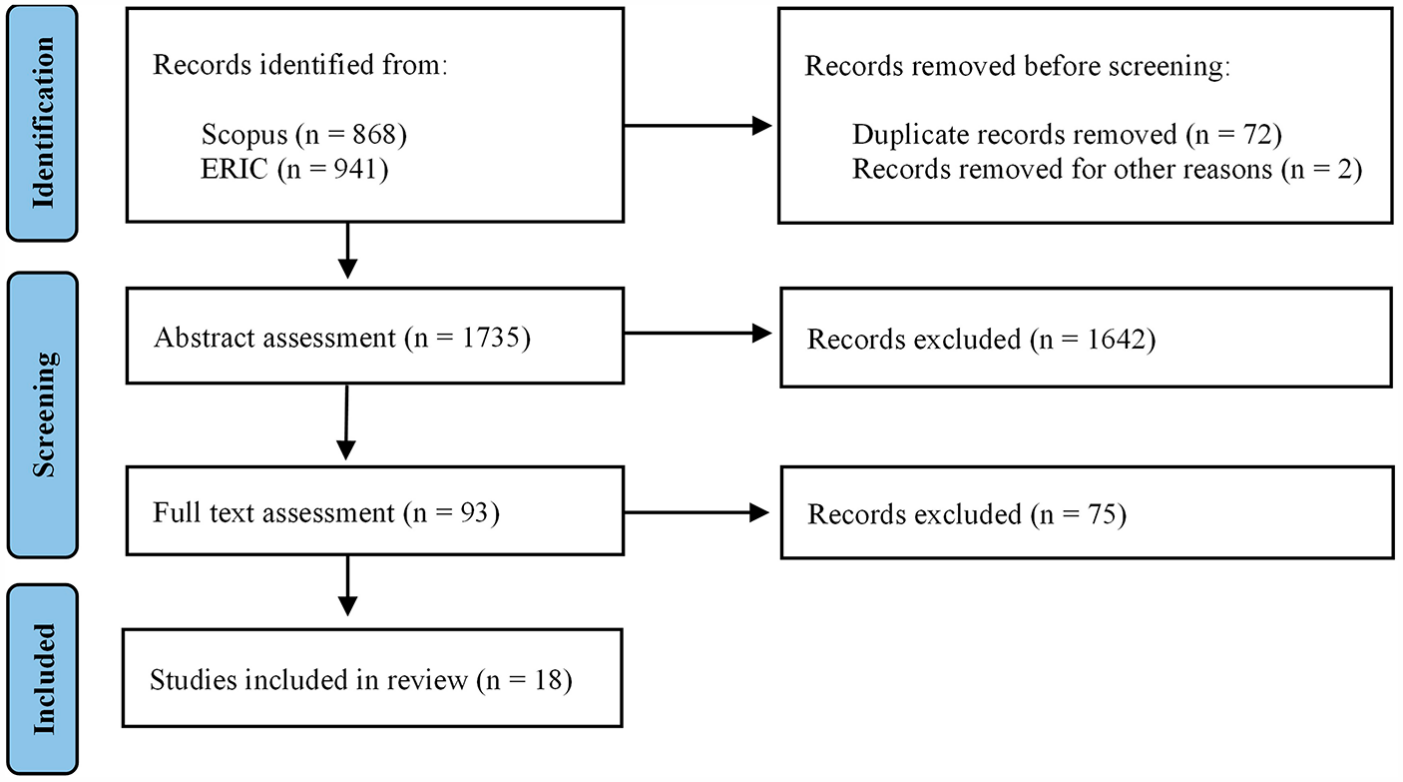
Flow chart of the identification and screening process

The search for relevant records was conducted in August 2022 in the scientific databases ERIC and Scopus, which were selected due to their focus on education research and broad interdisciplinary coverage, respectively. Due to the scope of the review, only peer-reviewed studies published in English between 2020 and 2022 were included in the search. Querying the selected databases resulted in a total of 1809 records, from which 72 duplicates and two retracted items were removed. The remaining 1735 records were then subjected to a screening process conducted by four researchers in which first the abstracts then the full texts of the papers were assessed. The four researchers ensured that the sorting processes were aligned through two initiatives: (1) the assessments were initiated by collaboratively assessing a number of articles, (2) another researcher went through papers that were marked with doubt by the researchers in order to make decisions about whether these papers should be included. Each study was assessed according to the following inclusion criteria: (1) the study must include empirical data collected between 2020 and 2022; (2) the study must focus on higher education; (3) the learning environment must have been characterized as student-centered prior to the pandemic; (4) the digital practices described must focus on the facilitation of the learning process (as opposed to other processes or content not related to the learning process). If a study did not meet the specified criteria, it was excluded from the review. However, if the necessary information was not clearly stated in the abstract, the researchers assessed the keywords and metadata during abstract screening, or searched the full text, to identify whether data was collected prior to the pandemic. In total, 1642 and 75 records were excluded based on the abstract and full text assessments, respectively. This resulted in a final pool of 18 studies eligible for inclusion in the review. Figure 2 displays the process of identifying and screening the record pool.

### Revisiting codes from case study

As mentioned above, the themes and questions emerging from our case study motivated a systematic literature review to explore whether these patterns have emerged in similar contexts across student-centered and collaborative learning environments, and to what degree. The findings from the literature review were used as a basis for revisiting the themes identified in the case study in order to explore the similarities, differences and nuances of emerging digital practices and competencies, and to contextualize these findings within a broader perspective. This process was systematized through qualitative tabulation, in which the themes and codes from the literature review constituted the structure and point of departure for revisiting the processed data from the case study using NVivo software. Each code from the review represented a row, and a corresponding column was dedicated to the case study data. We revisited the codes from the case study and inserted relevant inputs into the table in order to obtain a comparative overview of the data. The findings were then synthesized in the joint analysis presented in Sect. 3.2.

## Results

The final pool of eligible records was subjected to a mapping process in order to extract relevant information regarding authorship, year, country, research design, participants, learning model, activities, technologies, and competencies targeted by the learning design. Table 2 presents key information extracted from the 18 studies included in the review.

**Table 2:** Included studies

Author	Year	Country	Research design	Primary learning model	Targeted competencies
Alkhowailed et al.	2020	Saudi Arabia	Quantitative	PBL	Research skills and technical competencies
Cho & Kim	2021	South Korea	Quantitative	Flipped learning	Self-directed learning
Conde et al.	2021	Spain	Mixed	PBL	Teamwork
Garay-Rondero et al.	2021	Mexico	Mixed	Competency-based education, challenge-based learning, and experiential learning	Ethical argumentation, diagnoses an organizational process, designs an improvement situation for an organizational system or process, commitment to sustainability
Hauck & Melle	2021	Germany	Mixed	(Mobile) computer-supported collaborative learning	Collaboration
Jaiswal et al.	2021	USA	Mixed	PBL	Teamwork
Kalmar et al.	2022	The Netherlands	Mixed	Collaborative learning	Communication, cooperation, and empathy
Latorre-Cosculluela et al.	2021	Spain	Quantitative	Flipped classroom	21st century skills
Logemann et al.	2022	International	Mixed	Experiential and collaborative learning	Digital communication and intercultural competencies
Barbalho et al.	2021	Brazil	Mixed	PBL	Problem-solving
Morsi & Assem	2021	Egypt	Mixed	Collaborative learning	Digital competence
Ota & Murakami-Suzuki	2022	Japan	Mixed	PBL	Global awareness, problem-solving and finding, critical thinking and multicultural communication and understanding
Rachman et al.	2022	Indonesia	Mixed	Mobile-enhanced collaborative learning	Oral presentation
Ramachandran et al.	2021	USA	Mixed	PBL	Scientific literacy, critical thinking, problem-solving, and collaboration
Ripoll et al.	2021	Spain	Mixed	Cooperative learning	Transversal skills
Rook & McManus	2020	Australia	Quantitative	Non-placement work-integrated learning	Responsible leadership
Sá & Cruz	2021	Portugal	Quantitative	Active learning (using game-based learning activity)	Communication
Velaora et al.	2022	Greece	Quantitative	Gamification and PBL	Self-learning, problem-solving, teamwork

### Narrative synthesis

Following the mapping, the team of researchers conducted an inductive thematic analysis by coding the findings and topics of discussion of each study, resulting in 10 individual codes. These were condensed into four overall themes relating to the effects digital practices have on students’ interaction with teachers, other students, and content, as well as the competencies these types of interactions require and/or incite according to the study. Table 3 displays an overview of these codes and themes. The following section presents a narrative synthesis of key insights related to each theme.

**Table 3:** Themes and codes

Theme	Codes
Student-teacher interaction	Classroom interaction
	Instructor role and skills
	Feedback and assessment
Student-student interaction	Classroom interaction
	Groupwork
	Formal versus informal setting
	Tools promoting collaboration and connectivity
Student-content interaction	Self-efficacy
	Self-directed learning
	Concentration
Student competencies and emerging digital literacy	

#### Student-teacher interactions

The interactions between teachers/instructors and students emerged as a prevalent theme in nine of the 18 studies included in the review, which highlighted critical factors in and barriers to plenary interaction during lectures, as well as the provision of feedback from teachers and supervisors during group work. Some studies addressed overall classroom interaction in relation to synchronous and plenary online modalities, such as open discussion during live streaming sessions, with which the students in one study reported an average level of satisfaction (Alkhowailed et al., 2020). In other studies, students reported positive experiences with the online environment, the professors’ performance, and the provision of opportunities to express opinions during class, as well as a high degree of individual interactions between instructors and students when answering questions (Barbalho et al., 2021; Garay-Rondero et al., 2021; Cho & Kim, 2021).

Furthermore, teachers attempted to maintain connectivity and belonging in their classes by using tools which support video conferencing as well as synchronous and asynchronous communication between lectures, while adapting further practices, such as personalizing their communications, to add empathy and behavioral engagement in an effort to build social presence (Logemann et al., 2022). Nevertheless, an exclusively online environment affords less room for informal feedback, while a face-to-face presence can stimulate the students to ask more questions (Kalmar et al., 2022). The preliminary results of one study highlight the absence of a shared process between groups and lecturers when conducting synchronous collaborative sessions using breakout rooms: teachers lack easy access to group conversations online, while in face-to-face settings aspects of improvement can potentially be identified by simply walking into the room and listening to conversations among students (Sá & Cruz, 2021). However, in Morsi & Assem’s (2021) study instructors emphasized that answering questions for each group in collaborative group projects with large numbers of students can be difficult and time consuming in face-to-face settings, whereas asynchronous online provision of feedback provides an opportunity for detailed and documented feedback and allows problematic areas in the project report to be formatively highlighted.

In student-centered learning environments, the role of the instructor becomes that of a facilitator and advisor, and it has been demonstrated that this can be more efficiently achieved in face-to-face settings (Cho & Kim, 2021). One study suggests that during the rapid transition to an exclusively online environment and emergency remote teaching this role was further expanded to encompass the roles of coach and mentor, providing compassion to students as well as flexibility, understanding, and empathy (Logemann et al., 2022). However, as pointed out by Ota and Murakami-Suzuki (2022), online modes of instruction demand that teachers and instructors pay more attention to the individual student than is necessary when using face-to-face modes, and digital means of reaching students should thus be carefully considered.

#### Student-student interactions

Common for the studies included in the review is seeing interactions between students as a fundamental pillar of active and collaborative student-centered approaches. These interactions have received substantial attention from 10 studies, of which several have reported successful experiences with collaborative learning during the pandemic (Hauck & Melle, 2021; Jaiswal et al., 2021; Kalmar et al., 2022; Rachman et al., 2022; Rook & McManus 2020). The results demonstrate various benefits of online collaborative learning: it significantly enhances student participation (Rook & McManus, 2020); helps shy and introvert students participate more actively (Cho & Kim, 2021; Rachman et al., 2022); increases transversal competencies including communication, cooperation, and empathy (Kalmar et al., 2022); and improves team effectiveness and bonding (Jaiswal et al., 2021).

During the transition to fully online environments, collaboration and communication between students was commonly encouraged through the use of tools supporting both synchronous and asynchronous communication, file sharing, and learning management systems (LMSs), as well as video conferencing (used in 16 out of 18 studies in the review). In one study, breakout rooms, polling, and annotation features were emphasized to create an inclusive setting in which isolated students could work collaboratively (Ramachandran et al., 2021). Online collaboration tools were used to build a sense of togetherness and co-presence, and to compensate for the lack of in-person interactions (Logemann et al., 2022; Jaiswal et al., 2021). Furthermore, one study reports that online tools enabled better and more efficient collaboration compared to face-to-face settings, indicating that online tools may have caused students to be more diligent in their communication. However, improved efficiency in this regard cannot definitively be ascribed to the online mode of instruction (Jaiswal et al., 2021).

On the other hand, other findings suggest that motivation and team productivity was impeded in the online environment due to the lack of informal interactions and discussions among group members, ultimately resulting in a shift toward cooperation rather than collaboration (Kalmar et al., 2022). The absence of informal interactions is also addressed by Ota and Murakami-Suzuki (2022), who suggest that it is difficult for students to share moments prior to or after the formal online class, as they would in a conventional offline class. In addition, it can be challenging in online environments for group members to make the initial connections which allow them to establish relationships and trust. Furthermore, the limited presence of non-verbal communication online, especially in relation to asynchronous communication, has been found to afford more miscommunication in team meetings, ultimately making socio-emotional communication more challenging (Kalmar et al., 2022).

Although social and emotional connection may be difficult in an exclusively online environment, some studies indicate that the extent of the problem may be related to certain factors linked to the dynamics and demographics of the group. For instance, Logemann et al. (2022) found that teams with a high level of belonging reported more positive experiences with building co-presence on an online collaboration platform, reflecting positive relationships and solidarity in relation to their team dynamics. In addition, some studies emphasize that teams benefited from face-to-face meetings and interactions prior to the shift to exclusively online collaboration (Jaiswal et al., 2021), and groups which had existed before the lockdown had greater potential to succeed when working in an exclusively online environment than newly-formed teams did (Kalmar et al., 2022). Another key factor in the success of online teamwork may relate to students’ previous experiences with teamwork and the stage of education that they have reached. As Jaiswal et al. (2021) point out, teamwork skills develop over time, and team effectiveness has therefore been found to improve as students spend more time with one another over the semester. Consequently, remote learning might impede effective teamwork when this skill is yet to be developed by students (Conde et al., 2021).

#### Student-content interactions

Student interactions with learning content such as materials and learning activities were identified as a recurrent theme in 13 studies included in the review, indicating a broad variety of different modalities and emerging digital practices. Findings indicate that a student-centered and self-paced learning approach offers increased flexibility (Sá & Cruz, 2021) and enables students to work at their own pace and control their progress through the affordances provided by, for example, interactive videos and quizzes (Hauck & Melle, 2021). The asynchronous delivery of learning content allows students to view videos at any time; one study suggests that this can result in fewer requests for teacher support (Ripoll et al., 2021). In addition, synchronous activities may also yield benefits in online environments, as findings from one study suggest that working online may improve student motivation and focus by alleviating distractions such as side-chats (Morsi & Assem, 2021). However, distractions in online environments, such as games, YouTube videos, or other content that pops up on students’ screens, may also impact student concentration (Cho & Kim, 2021). This issue can be accommodated through interactive elements that keep students engaged, minimizing the risk of them zoning out (Hauck & Melle, 2021).

Despite several benefits, interactions with learning materials and the environment have not been seamlessly integrated into the emergency online teaching prompted by the Covid-19 crisis, and some of the studies address the challenge of transferring certain activities and learning experiences to online modes; one example is hands-on training in practical and clinical settings (Alkhowailed et al., 2020). In addition, several studies emphasize technology-related limitations, such as overloaded learning management systems, software-related constraints, frustration with technology among students, along with technical issues including sound failures, problems with memory availability, and unstable internet connections (Alkhowailed et al., 2020; Hauck & Melle, 2021; Kalmar et al., 2022; Rachman et al., 2022; Ripoll et al., 2021), which ultimately disrupt the learning process.

#### Student competencies in the ‘new normal’

**Fig. 3:**
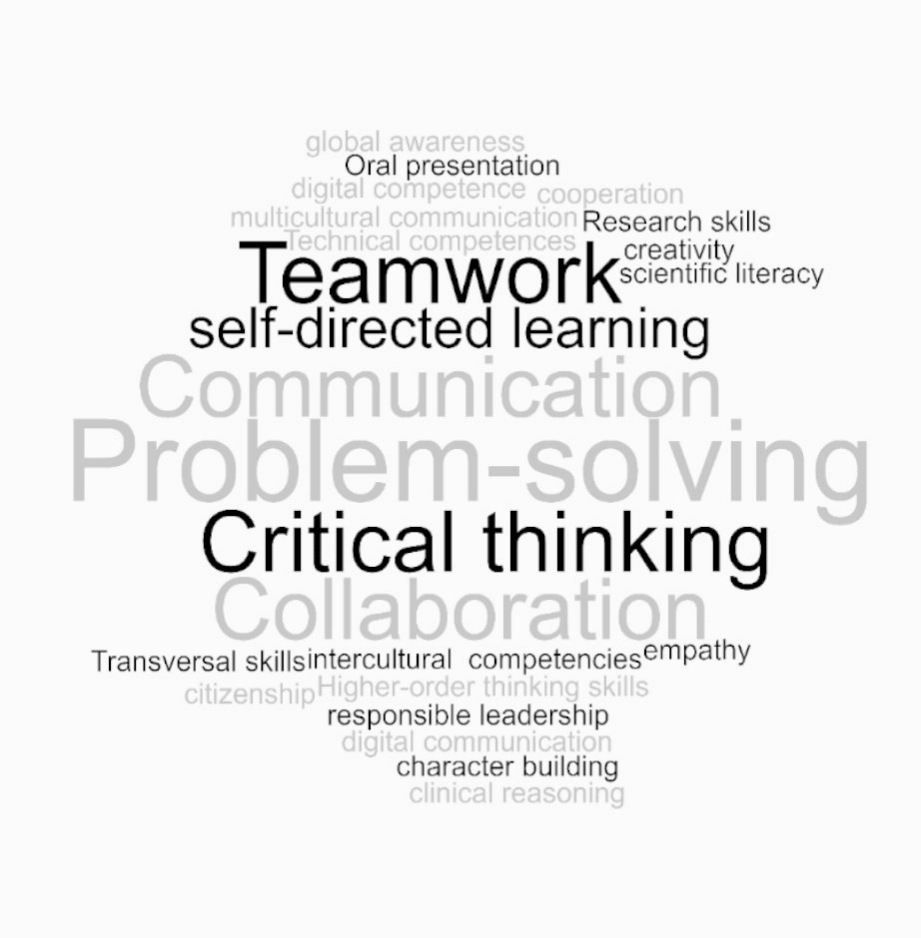
Explicitly targeted competencies across studies included in the review

As mentioned earlier, we were particularly interested in how emerging digital practices are guiding the shift into a ‘new normal’ and what competencies this shift requires and/or incites according to the studies. Thus, all the studies included in the review address competency development in varying degrees, with a substantial focus on literacy as a prevalent theme throughout the thematic analysis. Figure 3 displays a visual representation of the frequency of competencies explicitly targeted by the online learning interventions among the studies included in the review.

A notable pattern across the studies is the aim of supporting students in the development of transversal skills, such as teamwork and collaboration, which was identified in a total of seven studies. Another prevalent social skill is communication, which was identified in a total of five studies, of which one study specifically targets digital communication (Logemann et al., 2022). In general, social and transversal skills have received extensive attention among the studies, whereas other examples include cooperation, intercultural competence, multicultural communication and citizenship.

Despite the challenges presented by the pandemic, online collaborative learning has been found to help students acquire key competencies and autonomy (Ripoll et al., 2021), while the acquisition of transversal skills related to emotional connections and empathy has been found to help students combat the negative effects of social distancing during the pandemic (Logemann et al., 2022). The teaching and practice of social skills is central to the future development of online courses based on collaborative learning (Kalmar et al., 2022). As such, lessons learned from the pandemic, which will gain further relevance in the emerging hybrid workplace, should increase the attention to the development of social and transversal skills in higher education (Kalmar et al., 2022; Logemann et al., 2022).

Several studies also addressed the potential of blended, hybrid, or inverted learning approaches in a post-pandemic era (Latorre-Cosculluela et al., 2021; Ota & Murakami-Suzuki, 2022; Kalmar et al., 2022). These approaches may support students in developing skills such as creativity, critical thinking, communication and collaboration, and improve their self-efficacy by developing their self-directed learning and self-learning skills (Latorre-Cosculluela et al., 2021; Cho & Kim, 2021; Velaora et al., 2022). The online component of the blended learning experience can provide readily available and personalized online teaching materials and learning trajectories while facilitating collaboration, problem-solving, and reflection. Beyond the online components of blended learning, the planning of physical on-campus sessions may strengthen the sense of community and increase unplanned socio-emotional interactions and peer feedback (Kalmar et al., 2022). Furthermore, knowledge acquisition and knowledge sharing can be supported by a combination of asynchronous and synchronous online learning activities (Ota & Murakami-Suzuki, 2022).

### Joint analysis: Revisiting the case study through the lens of the literature

In the following sub-sections, the findings from the literature review will be used as the basis for analysis of the empirical data collected in the early reactive case study and comparison of findings related to the identified themes, highlighting similarities, differences and nuances that might further inform the transition into online, hybrid, and blended modes of student-centered learning. In this analysis, we group findings into two overarching themes based on the four themes identified in the literature review: (1) emerging digital practices, i.e. student interactions with teachers and supervisors, content, and each other; and (2) emerging digital literacy, i.e. the development of competencies among students and teachers that are relevant to this ‘new normal’ in higher education.

#### Emerging digital practices: interactions revisited

The literature review above has emphasized the emergence of new digital practices supporting student interactions with teachers, content, and one another during ERT in student-centered learning environments. Prior to the pandemic, these interactions mainly took place in classrooms and lecture halls, in group rooms and collaborative workspaces, as well as in individual settings, which have all been substantially disrupted due to the alternating confinement measures imposed by the pandemic. Despite these measures, studies in the review indicate that teachers have supported student-teacher interactions by experimenting with digital practices supporting connectivity, belonging, social presence (Logemann et al., 2022), by situating the interactions in distributed online spaces supported by means of existing technologies. The emergence of these practices has similarly been observed in the case study, in which teachers experimented with video conferencing platforms and both synchronous and asynchronous communication tools in and between lectures, along with online collaborative whiteboards, class timing and duration, quizzes and polls, class sizes, the combination and mixing of classes, online seminars, chat communication, and learning analytics for differentiation based on the needs of learners. Thus, in spite of lockdowns and restrictions, teachers in the case study managed to maintain a certain level of social interaction and student engagement in the digital space.

Experimental and reactive digital practices have similarly been adapted to support interactions between students and build togetherness and co-presence, as well as to compensate for the lack of in-person interactions (Logemann et al., 2022; Jaiswal et al., 2021) through online collaboration tools. However, as with other studies in the literature review (Kalmar et al., 2022; Ota & Murakami-Suzuki, 2022), teachers and students in the case study both emphasized the absence of informal learning and situated knowledge-sharing both among students and groups and between students and teachers that would usually take place spontaneously in-between lectures or meetings in a physical setting. As such, the overall experience with these reactive practices is mixed; while they inevitably contribute to a reduction of issues related to social well-being and isolation due to physical restrictions, new issues have emerged in relation to the quality of social interactions being affected by the absence of, for example, non-verbal cues, informal discussions, and socio-emotional communication (Ota & Murakami-Suzuki, 2022; Kalmar et al., 2022), which serve a critical role in collaborative student-centered environments.

However, as outlined in the literature review, the extent of these issues may be relative to certain group dynamics and demographics, such as the level of belonging felt by the students and the balance between face-to-face and exclusively online forms of collaboration, or whether the group existed prior to lockdowns (Logemann et al., 2022; Kalmar et al., 2022; Jaiswal et al., 2021). This is confirmed by data from the case study: supervisors reported more difficulties in groups in earlier semesters compared to later semesters, suggesting that this was due to smaller groups and more developed collaboration skills in the later semesters. This variation in students’ ability to collaborate was highlighted both in the review and in interviews with teachers, who argued that while project work ensured a certain level of both social and academic interaction between students during lockdowns, the quality of collaboration varied greatly according to the degree to which the students had advanced in their education. According to the teachers, students in their first year and groups that were formed after lockdown demonstrated noticeably more individualized patterns and more instances of miscommunication and conflict, while groups in later semesters did not exhibit these issues to the same degree. As one teacher stated:

My experience supervising was that it [project work] was one of the things that worked well. But it should be noted that I supervised Bachelors students [6th semester] and Masters students [10th semester] in the spring, and those students obviously had the ‘social putty’ already (FOC1/TE2)

Students in larger groups also noted that interactions were significantly impeded in online meetings with many participants, while meetings with fewer participants or one-to-one meetings conversely supported interaction during lockdowns. During group work, short online meetings were also used to supplement face-to-face meetings when students worked in distributed locations. Different phases of project work required different modes; when starting or finishing a project, face-to-face meetings were preferred for brainstorming ideas, social bonding, and discussion (collaborative work), while writing and simple tasks could be completed with mainly online meetings (cooperative work). One student noted that:

Idea generation works best if you are face-to-face, because then you have the possibility to interrupt each other. The writing process works fine online. The final phase with revisions, etc., works better if you sit together in real life (FOC5/ST1)

Kalmar et al. (2022) observed that exclusively online teamwork shifted toward a cooperative distribution of the workload rather than students collaboratively solving problems together, which may explain why students felt that online teamwork was less effective. Thus, as pointed out by the student quoted above, project- and problem-based teamwork can beneficially be distributed through a blend of online and physical spaces, as certain stages involving collaborative activities may profit from face-to-face meetings, which can be supplemented by individual and cooperative activities online.

In a similar vein, both students and teachers in the case study agreed that a 1:1 transfer of analogue face-to-face pedagogy to an online setting was generally unsatisfactory, and preferred flipped or blended approaches to *teaching*. For teachers, the lack of social and emotional response (affect) from students can contribute to an experience of lack of control and insight into the students’ learning process. Similarly, Sá and Cruz (2021) highlighted the absence of a shared process between groups and lectures in synchronous collaborative sessions. In flipped and blended approaches, which mainly included preparatory work such as watching pre-recorded lectures, short video lectures, and doing exercises, teachers in the case study experienced increased interaction with students during class. However, it was noted that students struggled with transitioning to a flipped classroom and preferred synchronous online lectures, which they were somewhat accustomed to:

(…) I think they need training to accept this [flipped classroom] teaching form. That preparation is required … is the principle of flipped classroom; that they have prepared before we meet. And I think they find that hard to swallow and it will likely be a long haul (FOC7/TE4).

However, preparatory work consisting of different digital teaching resources offers valuable repositories for students to revisit in the periods leading up to exams, thus making student interactions with content more flexible, continuous, and integrated into the entire learning process, including exams. Similarly, data on video views in Hauck and Melle (2021) indicated that many students returned to the interactive videos to work on them a second time when preparing for exams toward the end of the semester.

#### Emerging digital literacy: student and teacher competencies revisited

As described in the previous sections, student-centered learning environments have taken new forms for both teachers and students during the pandemic and the various lockdowns. Under the pressure of changing conditions, new digital practices have emerged at all levels of education and for all roles, from individual student work to group work, from simple course activities to the structuring of semesters.

For teachers in the case study, a transition from ERT was initiated at the very beginning of the pandemic, when teachers simply uploaded slideshows with voiceovers or conducted lectures through online video conference calls, and led to further experimentation, with models such as flipped classrooms and blended learning emerging as the most common models. At the student level, problem-based project work has afforded experimentation with different forms of group work, i.e. distributed vs. face-to-face, synchronous vs. asynchronous, cooperative vs. collaborative modes, and innovative combinations of all of the above, affording new not only digital but also transversal competencies:

*All the students [in a robotic sailing project] were working from home and part of the project then became to produce a technical solution that would allow them all to work on this boat, and the computing side of the boat remotely. So, they had to completely change their project because there’s only one person who had access to the boat and she was kind of being remote controlled by the others to do all the tests on it. They were telling her what to do and so on. And then they actually created this new thing. So, I think we should take the opportunity to increase our skills and our knowledge about how to deal with these situations and attack those problems, too. And they can be the topics of interesting projects, actually (FOC13/TE3).*

This reflects competencies among teachers and students alike to better integrate multi-modal forms of working. These options were also available prior the pandemic, but were not experimented with to the same extent as they were during the lockdowns. In traditional educational settings, when classrooms and labs were not accessible during ERT, students’ experience of motivation, self-efficacy, and cognitive engagement decreased *(*Aguilera-Hermida 2020), whereas in this case study teachers and students alike experienced high levels of student agency and self-efficacy:

*I was quite impressed with the creativity they showed with regard to finding [project] themes, that were trending right now. In that moment, I was happy and a little proud to be working at a university that actually forces the students to think in and with time. And it became exemplary learning in the way, that they went out and did something in the world, for the world, while it was happening (FOC8/TE1).*

In a systemic PBL environment, students have considerable responsibility to set goals, organize their own time and learning activities, and be reflective about these processes. As such, teachers and supervisors reported big differences in students’ self-efficacy levels, with younger and academically weaker students still learning the skills necessary to perform project work, struggling when facing new challenges, and requiring more pedagogical supervision and support from their supervisor. On the other hand, experienced and capable students in the higher semesters exhibited excellent adaptability in recontextualizing project ideas and plans, given the ongoing crisis. However, the supervisors’ competence (and time) to facilitate and assist this transition was instrumental to its success:

*In my study program, we couldn’t do the lab work we had planned to do. Instead, some groups were allowed to make their project results in a theoretical form, but my group was lucky to have a flexible supervisor, who gave us the option to write about corona. We saw this as an opportunity to learn a lot in a new way (…) It was a good supervisor, who was flexible, replied to our emails and was easy to contact (FOC3/ST4).*

Supervisors also reported that more extroverted and socially-oriented learners struggled to maintain motivation and discipline, whereas more introverted and individually cognitive-oriented learners seemed to experience more positive effects from online participation, and exhibited a higher degree of self-efficacy. Similarly, studies in the literature review report that online collaborative learning led shy and introvert students to become more active participants (Cho & Kim, 2021; Rachman et al., 2022). In the case study, students that were technically adept and had experience in participating in online communities adapted to the situation by creating new project-related online groups and networks: in this case, digital competence helped support transversal and collaborative competencies, and vice versa.

## Discussion

The analysis above highlights the emerging practices adapted in a crisis-prompted state to ensure continuity of education in student-centered learning environments. The following section will discuss the main findings in light of contemporary research and literature, and will address the implications for research and practice which qualifies and informs the ongoing transition into a ‘new normal’ for student-centered higher education.

Many studies, both in student-centered and traditional learning environments, emphasize the need to distinguish between emergency remote teaching/learning experiences instituted during the pandemic and established online teaching and learning (OTL) practices (Aguilera-Hermida, 2020; Rapanta et al., 2021; Hodges et al., 2020; Lee et al., 2022). The latter practices originate from careful consideration of instructional design and planning and are often associated with flexibility of teaching in time and space (Hodges et al., 2020), paying particular attention to teachers’ readiness for the transition to OTL (Scherer et al., 2021), and which digital spaces are compatible with and integrated into students’ daily lives (Conde et al., 2021). Thus, it is likely that the most prevalent issues encountered during ERT in student-centered environments would have taken different forms if the teachers had time to carefully consider the learning design in accordance with the alternating modalities of the pandemic period. More organizational focus and support in the ‘new normal’ will support the development of OTL and avoid many pitfalls and barriers experienced during ERT. These experiences, along with emerging digital practices, will feed into and further inform the ‘new normal’ in higher educational practices.

As indicated in our empirical study, one-to-one transfer of face-to-face pedagogy in online classrooms is unsatisfying for both students and teachers. Nevertheless, this transfer has often been a default strategy in the temporary and crisis-prompted shift into ERT, leaving instructors with little time to prepare alternative delivery modes to remote solutions that would otherwise have been delivered face-to-face (Hodges et al., 2020; Lee et al., 2022); this results in mostly synchronous modalities as well as teacher-centered knowledge transmission (Lee et al., 2022; Crawford et al., 2020). Although the contextual factors surrounding the studies included in the review and in our own empirical study are inherently student-centered, the pattern of teacher-centered synchronous modalities has also been observed in relation to ERT during online lectures and plenary activities. Some of these unsatisfactory experiences, as shown in the literature review and case study, will most likely not translate into the ‘new normal’ for higher educational practices.

However, these activities have been supplemented with a high degree of student-centered, collaborative activities, which were experienced as a fundamental strength during periods of confinement. While students and teachers still generally prefer face-to-face classroom settings, the systematic review and case study suggest that student-centered learning models such as PBL were generally considered strengths during the early and rapid transitions to online learning during the pandemic, and in combination with new digital practices and teachers’ facilitation of the development of student competencies and digital literacy.

The lessons learned from crisis-prompted adaption of student-centered education to online and blended modalities have three main implications for research and practice. First, while the critical factors and barriers to social interaction have received extensive attention in the studies included in the review, these studies also center the aim of supporting students in developing primarily social and transversal skills and competence in digital environments, emphasizing a need for the further development of digital literacy in online student-centered environments among students and teachers alike. However, further research is required to develop methods to reconstruct and increase socio-emotional interactions among teachers and students in these settings (Kalmar et al., 2022; Cho & Kim, 2021; Latorre-Cosculluela et al., 2021), as well as on teacher professional development (Englund et al., 2017; Kalmar et al., 2022).

Second, the prerequisites for maximizing the benefits of online collaborative learning include adequate digital infrastructure, open access to computer equipment, stable internet connections, and the training of both teachers and students in the use of targeted collaborative platforms (Morsi & Assem, 2021; Rachman et al., 2022; Latorre-Cosculluela et al., 2021). These implications serve as an important reminder in the design of exclusively online environments. Third, as we transition ‘back’ into a post-pandemic world without physical restrictions, it has become crucial to consider how to continuously facilitate the necessary pedagogical innovations and emerging digital practices introduced in a crisis-prompted state of emergency. It is important to conduct longitudinal and cross-contextual studies to generate insights into the long-term impacts of digital transformation on student learning and digital literacy in order to further a qualified and informed transition ‘forward’ into a ‘new normal’ of post-pandemic higher education that supports student-centered learning and competency development.

As the world shifts into a post-pandemic state with other escalating global challenges, the potential of inverted, flipped, blended, and hybrid modalities has been emphasized in the studies included in the review, as well as in our empirical study, indicating a future pattern of a mixture between online and campus-based face-to-face teaching and learning practices in student-centered learning environments. In a similar vein, the binary distinction between exclusively online and exclusively face-to-face learning activities has been challenged in recent years, as scholars argue for a post-digital perspective that sees learning situations as complex entanglements of people, spaces, activities, and material, in which the digital and non-digital are intrinsically and inextricably interconnected (Networked Learning Editorial Collective (NLEC), 2021; Green et al., 2020; Bayne et al., 2014). Thus, while online, blended, and hybrid learning might be used as terms to differentiate between design scenarios, this distinction might not capture the complexity and intricate connectedness of students’ interactions with each other and with learning tasks, teachers, and content co-present in multiple spaces (Jandrić & Boras, 2015; Fawns 2019; Networked Learning Editorial Collective (NLEC, 2021).

## Conclusion

This paper has investigated the emerging digital practices which have been adapted to support student-centered learning environments in higher education since the beginning of the pandemic. This investigation has also explored the emerging digital literacy that these new practices will require and incite. Motivated by the findings of our own reactive study signifying emerging digital practices and the relevance and prevalence of certain transversal competencies in relation to student resilience, adaptability, self-directed learning, and interpersonal competencies, our aim was to explore whether and to what degree these patterns have emerged in similar contexts characterized by student-centered educational approaches.

By synthesizing the experiences disseminated in 18 early reactive empirical studies, our findings highlight emerging practices supporting students’ interaction with teachers, content, and one another during ERT in student-centered learning environments. In general, these interactions have been situated in distributed online spaces supported by existing technologies, and both teachers and students have demonstrated a high level of adaptability and creativity by experimenting with modalities to support core learning activities. Although central social and collaborative aspects of student-centered learning environments have been highlighted as a particular strength during periods of lockdown and social confinement, new issues have emerged in relation to the quality of mainly student-student interactions and collaboration in exclusively online environments, resulting in individualization of project work, especially for younger students and newly formed groups, although project groups were generally found to be open to, and successful in, experimenting with emerging digital practices for collaboration.

Thus, in spite of critical factors and barriers to social interactions highlighted in both the case study and the literature review, the aim of supporting students in the development of social transversal competencies in digital environments has been brought to the fore, emphasizing the need for the further development of digital literacy in online student-centered environments in the future—among students and teachers alike.

Although this study offers a snapshot into the complex landscape of a practice characterized by rapid and constant transformation, these findings may be considered indicative of existing patterns that have been further accelerated and amplified through the experience of the pandemic. However, the main limitation of this research is that both our empirical case study and the early reactive studies included in the review were conducted in a crisis situation, in which practitioners had little time to carefully prepare for the disruptive and rapid shift in modalities and instructional designs. As such, if this study were to be replicated for more established OTL practices, it is likely that the results would be different. Furthermore, the literature review only included studies published between 2020 and August 2022. With the world shifting in and out of lockdowns and social restrictions, it is likely that a future review including studies published in the coming years will be characterized by more nuanced experiences with online and blended approaches to supporting student-centered learning environments and competency development.

## Data Availability

The datasets generated during and/or analysed during the current study are available from the corresponding author on reasonable request.
